# AqUavplant Dataset: A High-Resolution Aquatic Plant Classification and Segmentation Image Dataset Using UAV

**DOI:** 10.1038/s41597-024-04155-6

**Published:** 2024-12-20

**Authors:** Md. Abrar Istiak, Razib Hayat Khan, Jahid Hasan Rony, M. M. Mahbubul Syeed, M. Ashrafuzzaman, Md. Rajaul Karim, Md Shakhawat Hossain, Mohammad Faisal Uddin

**Affiliations:** 1 RIoT Research Center, Independent University, Bangladesh, Dhaka 1229 Bangladesh; 2 Department of Computer Science and Engineering, Independent University, Bangladesh, Dhaka 1229 Bangladesh; 3 Department of Crop Botany, Bangladesh Agricultural University, Mymensingh, 2202 Bangladesh

**Keywords:** Agriculture, Environmental impact

## Abstract

Aquatic vegetation species are declining gradually, posing a threat to the stability of aquatic ecosystems. The decline can be controlled with proper monitoring and mapping of the species for effective conservation and management. The Unmanned Ariel Vehicle (UAV) aka Drone can be deployed to comprehensively capture large area of water bodies for effective mapping and monitoring. This study developed the AqUavplant dataset consisting of 197 high resolution (3840px  × 2160px, 4K) images of 31 aquatic plant species collected from nine different sites in Bangladesh. The DJI Mavic 3 Pro triple-camera professional drone is used with a ground sampling distance (GSD) value of 0.04-0.05 cm/px for optimal image collection without losing detail. The dataset is complemented with binary and multiclass semantic segmentation mask to facilitate ML based model development for automatic plant mapping. The dataset can be used to detect the diversity of indigenous and invasive species, monitor plant growth and diseases, measure the growth ratio to preserve biodiversity, and prevent extinction.

## Background & Summary

Aquatic plants represent a specialized category of flora adapted for survival and growth in aquatic environments such as freshwater lakes, rivers, ponds, and marine ecosystems^1^. These plants are notable for their contribution as primary producers of oxygen via photosynthesis^2^. They are vital to various domains including human medicine^3^, food^4^, fodder^5^, ornamentation^6^, phytoremediation^7^, and agriculture, highlighting their diverse roles in rural economies and environmental stewardship^8^. However, there is a gradual decline in aquatic vegetation species, posing a threat to the stability of aquatic ecosystems^9^. To mitigate this ecological disruption, it is crucial to conduct thorough surveys and mapping of aquatic vegetation species for effective conservation and management^10^. Precise mapping supports ecological research by providing valuable data on plant distribution, biodiversity, habitat condition, and ecosystem dynamics^11^.

Traditionally, aquatic plant mapping is carried out by manual survey or using satellite-based remote sensing. However, accurate mapping and monitoring using these methods remain a challenge due to their inherent limitations^12^. Manual survey is an inefficient, labor intensive, and time consuming approach, therefore impractical for larger-scale applications. In contrast, satellite-based remote sensing can capture large-area imagery at the macro level but fails to capture micro-level details of aquatic plants with the required precision due to low spatial resolution. In addressing these challenges, the Unmanned Aerial Vehicle (UAV) or Drone equipped with cutting-edge multispectral camera systems, low altitude, long-range flight path with precision positioning has shown high potential^13^.

In recent times, few aquatic datasets have been developed using UAVs of different configurations that underscore the competency of UAVs to capture detailed imagery of aquatic plant species. A synopsis of the aquatic plant datasets produced using UAV is presented in Table 1. This table highlights nine datasets each described by the amount of images, number of aquatic plant species identified, UAV ground sampling distance (GSD) and total number of sites covered for data collection. The UAVs used in these studies are equipped with RGB^14^ or multispectral^15^ cameras. RGB cameras are handy for capturing high-resolution images and real-time transmission and processing, but lack detailed spectral information. Multispectral cameras can offer richer spectral data to complement RGB images for precise analysis; however, it requires specialized equipment and time-consuming offline processing^16^. Furthermore, the reported data sets are limited by the selection of high value of GSD that is directly proportional to the quality of images captured by the drone (the recommended value of GSD to obtain the best image quality with a RGB camera is ≤1*cm*/*px*)^17,18^. The numbers of aquatic plant species are also very limited for most of the datasets (less than 9) expect the one in^19^. Also, the number of locations / sites visited for data collection is limited, which raises concern about the diversity of the recorded species.

**Table 1:** Comparison of Datasets for Aquatic Plants.

Dataset Reference	Data Quantity	Total Sites	GSD (cm/px)	Total Species
Husson *et al*.^19^	200-300 objects/block	3	5.6	26
Chabot *et al*.^45^	3090 objects	5	13 (RGB), 4 (M.Spectral)	6
Husson *et al*.^24^	—	5	5	9
Husson *et al*.^46^	—	5	5	7
Abeysinghe *et al*.^47^	—	1	13.9	5
Brinkhoff *et al*.^48^	—	4	5	3
Marcaccio *et al*.^49^	—	1	8	3
Abreu *et al*.^50^	962 images	1	—	2
Taddia *et al*.^51^	65535 images	2	5, 8	3

To complement existing datasets and address identified limitations, this research introduces the *AqUavplant* dataset^20^. This dataset comprises 197 high-resolution images (3840px × 2160px, 4K) representing 31 aquatic plant species, sourced from nine different locations in Bangladesh. Image acquisition is performed using the DJI Mavic 3 Pro drone equipped with a triple-camera system, achieving a GSD of 0.04-0.05 cm/px to maintain high detail fidelity. Bangladesh encompasses 18,290 *km*^2^ (14%) of water-covered territory, significantly contributing to the biodiversity of aquatic plants^21^. The images have been annotated for binary semantic segmentation (distinguishing aquatic plant species from the background) and multiclass semantic segmentation (identifying each species and the background) using advanced annotation tools to support various machine learning (ML) tasks. Lastly, the usability of the dataset is demonstrated through benchmarking with different segmentation models.

## Methods

**Fig. 1:**
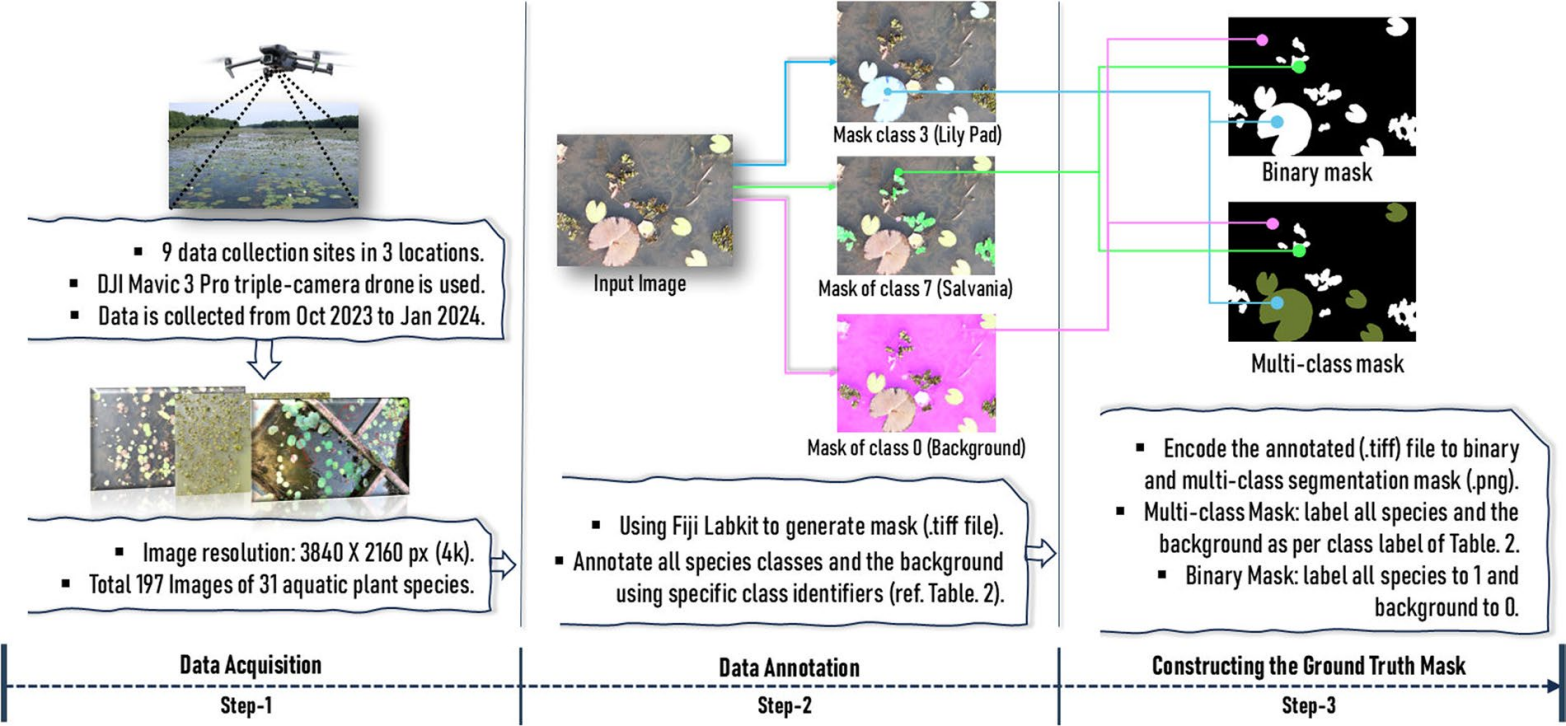
The step-by-step approach to prepare the dataset.

The methodology can be summarized into three steps, as shown in Fig. 1. In step-1, the experimental setup is done and data acquisition is conducted using the Drone. In Step-2, the data annotation is performed through the mask outline. Finally, the ground truth mask for semantic segmentation is constructed in Step-3 (Fig. 1).

### Study Area

**Fig. 2:**
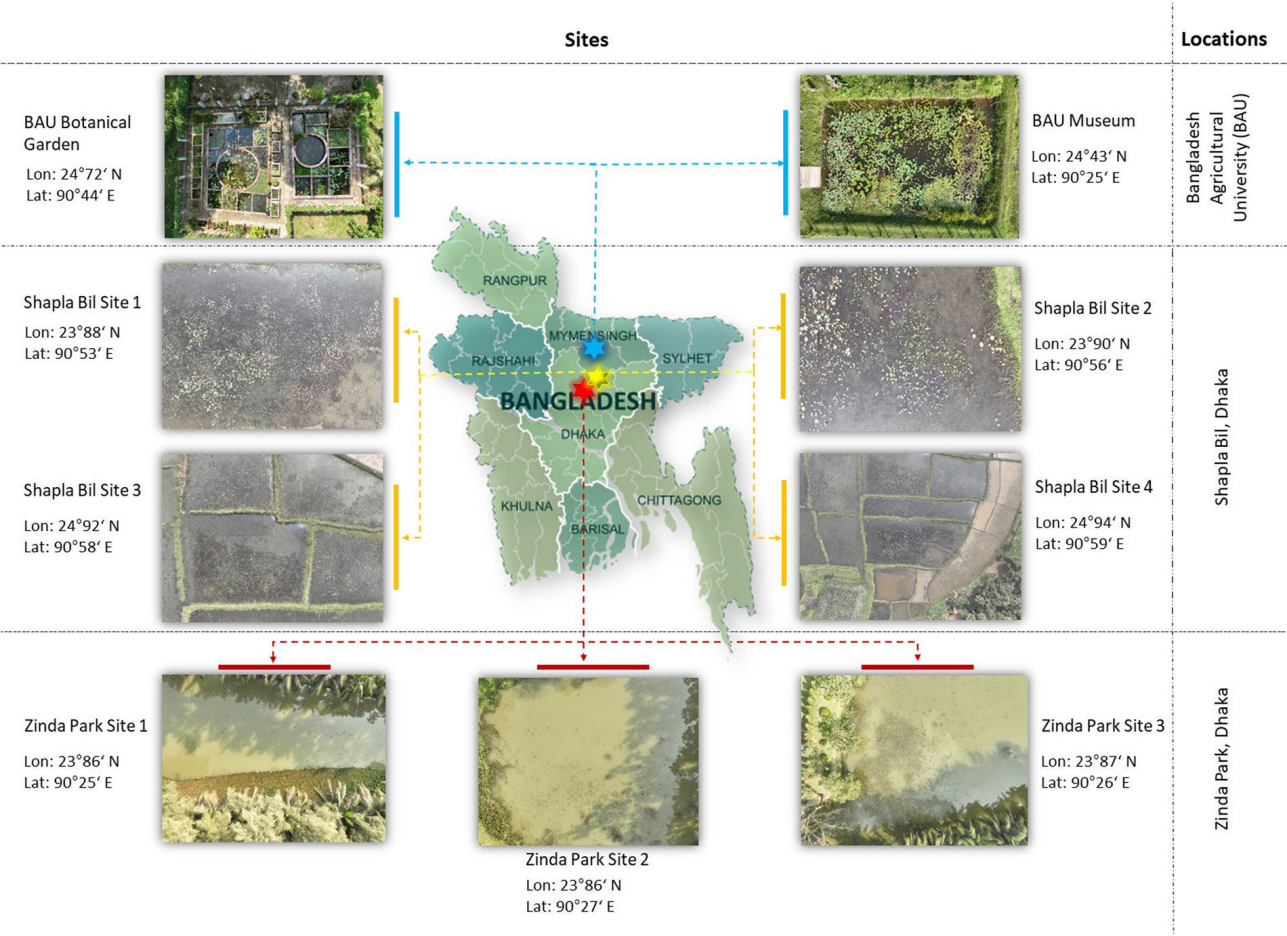
Image data is collected from 9 (Nine) sites at 3 (Three) locations in Bangladesh.

In Bangladesh, the most common types of freshwater environments are haor, baor, beel, lake, pond, rivers and floodplains that host various aquatic plants^22^. For this study, three locations are selected based on aquatic plant distribution and diversity, and ease of data collection using the UAV. These locations are: Bangladesh Agricultural University (BAU), Mymensingh, Zinda Park, Dhaka and Shapla Bill (in English the Water Lily Lake), Dhaka. BAU hosts the second largest botanical garden in Bangladesh considering the number of plant species that are collected from various parts of Bangladesh^8^. It is enriched with live collection and conservation of diverse plant species in aquatic habitats for educational and research purposes^8^. Zinda Park is an amusement park with 33 acres of land that has five natural lakes. Shapla Bill is a 22 acres of low waterland. Both locations have naturally grown aquatic plant species. Each of these locations are divided into multiple sites for efficient and comprehensive data collection. An exclusive summary of these sites is presented in Fig. 2.

### Data Acquisition

Image is captured using the DJI Mavic 3 Pro (DJI RC) drone^23^. Mavic Pro is a triple-camera drone equipped with a Hasselblad camera (4/3 CMOS, 20 MP) and dual tele cameras (1/1. 3^*″*^ CMOS with 48 MP and 1/2^*″*^ CMOS with 12 MP). For route planning and positioning, it uses GPS, Galileo and BeiDou satellite positioning systems. It also uses a 3-axis mechanical gimbal (tilt, roll, pan) for stabilization that generates very low angular vibration (±0.001° to  ± 0.005°). The following drone configuration is set for optimal image acquisition^19,24^, the Hasselblad camera is aligned downward and perpendicular to the cruise path. The camera resolution is set to 3840 px × 2160 px (4K). Other camera configurations, for example ISO, shutter speed, aperture, and focal length, are set to auto-mode to best adjust with dynamic variance of the environment (e.g., sunlight, shadow). The flight altitude is set at 2.5 m from the water level to ensure a GSD of 0.04-0.05 cm/px. The cruise speed is set to 1 m/s. These configurations are optimal for capturing best quality images without compromising the motion blur and exposure. In addition, it ensures that there is no vibration on the water surface from propeller thrust. To cover a site in auto-flight mode, a pre-planned route is set for the drone using waypoints marked on the map using the DJI flight app^25^.

Data acquisition spans four months, from October 2023 to January 2024, under ideal conditions characterized by bright sunlight and minimal or no wind. During drone flight, both images and motion videos are obtained in 4K resolution as previously described. Following data collection at each site, a thorough image analysis is performed to verify full site coverage. Any uncovered areas are supplemented using frames extracted from the motion video. This strategy ensures comprehensive site coverage and accurate species documentation; however, in a few cases, it introduces partial motion blur or exposure (approximately 12% of the images). These images pose a minimal threat to data quality, as application of the dataset in several segmentation algorithms shows promising results (discussed in the Technical Validation Section). Furthermore, such issues can be effectively reduced or eliminated with different restoration methods^26^. The resulting dataset comprises 197 images, capturing 31 unique aquatic species.

### Data Annotation

In this step, mask outlining is performed for each image to identify the classes of aquatic plants and the background for semantic segmentation. Multiple authors of this article performed the mask outlining annotation to support both binary and multiclass semantic segmentation. This task is performed in three stages. First, various aquatic species are correctly identified from the images and assigned unique numbers as a class identifier. The fifth author, a distinguished specialist in botany, performed this task. A total of 31 species are identified that are leveled with class numbers between 1 and 31. The background of the image is assigned to 0. Table 2 details the 31 aquatic plant class names, scientific names, class level number, and the location / site where the plants are found.

**Table 2:** Different plant labels and information.

Class Name	Scientific Name	Class Label	Location
Background	—	0	All
Water Iris	*Iris pseudacorus*	1	BAU
Keshordam	*Eclipta prostrata*	2	BAU, Shapla Bil
Lily Pad	*Nymphaea spp*	3	All
Water Mimosa	*Neptunia oleracea*	4	BAU
Lotus Leaf	*Nelumbo nucifera*	5	All
Water Popy Leaf	*Hydrocleys nymphoides*	6	BAU
Salvania	*Salvania cucullata/molesta*	7	All
Chinese Water Spinach	*Ipomoea aquatica*	8	BAU
Water Chestnut	*Trapa natans*	9	BAU, Shapla Bil
Arrowhead	*Sagittaria latifolia/montevidensis*	10	BAU
Sagor kolmi	*Ipomoea pes-caprae*	11	BAU
Water Plantain	*Alisma plantago*	12	BAU, Shapla Bil
Mexican Sword Lily	*Echinodorus palifolius*	13	BAU, Shapla Bil
Shola	*Aeschynomene*	14	BAU
Motmotey	*Lippia alba*	15	BAU
Floating pennywort	*Hydrocotyle ranunculoides*	16	BAU
Jhanji	*Ceratophyllum demersum*	17	BAU
Umbrella plant	*Cyperus alternifolius*	18	BAU
Chinese Coin Plant	*Pilea peperomioides*	19	BAU
Chechra	*Pistia stratiotes*	20	BAU
Sushni	*Marsilea minuta*	21	BAU
White Lily	*Nymphaea alba*	22	BAU, Shapla Bil
Duck Weeds	*Lemna minor*	23	BAU
Water Clover	*Marsilea quadrifolia*	24	BAU
Topapana	*Pistia stratiotes*	25	BAU
Mosquito Fern	*Azolla filiculiodes*	26	BAU, Shapla Bil
Kachuripana	*Eichhornia crassipes*	27	All
Mosaic Plant	*Ludwigia sedioides*	28	BAU
Cape blue lily	*Nymphaea capensis*	29	BAU, Shapla Bil
Panchuli	*Nymphoides indica*	30	BAU
Gechu	*Apanogeton natans*	31	BAU

Here, ‘All’ refers that the plant exists in all of the three locations.

**Fig. 3:**
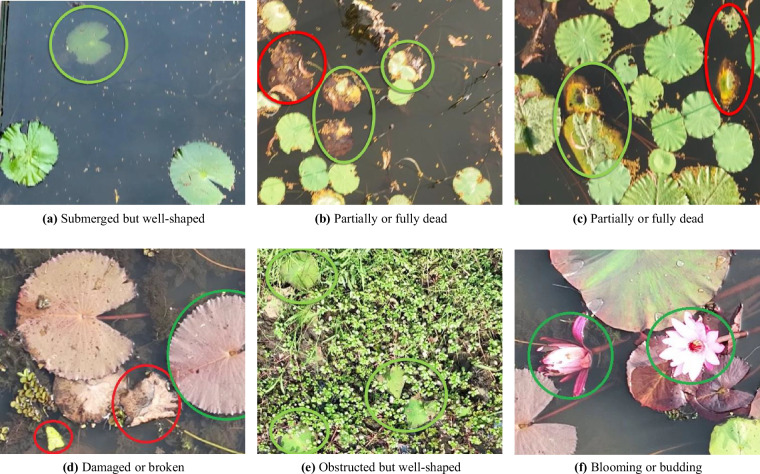
Example of some anomalous cases to include and exclude from classifying. Green encircled plants are classified, and red encircled are counted as background.

As aquatic plant images are captured in their natural habitat, identification of each plant requires resolution of inherent anomalies in the images. The anomalies are summarized in Fig. 3 based on which the following decisions are made to perform the mask outlining annotation. Aquatic plants with the following properties are considered for mask outlining, (a) plants that are floating or submerged in water, but the shape and texture are clearly identifiable (as in Fig. 3a), (b) plants that are partially dead or damaged or obstructed by other backgrounds but shapes are visually recognizable (green marked plants in Fig. 3b,c,d,e), and (c) plants that are in different stages of blooming and budding (Fig. 3f). Other variations, for example, dead, decomposed or broken plants that are visually unrecognizable (the red-marked plants in Fig. 3b,c,d) are excluded.

Finally, mask outline is performed using the Labkit^27^ plugin from Fiji toolKit (https://imagej.net/plugins/labkit/). Labkit is a user-friendly intuitive pixel classification plugin in Fiji for semantic segmentation (both binary and multiclass) of the image data.

### Constructing the Ground Truth Mask

**Fig. 4:**
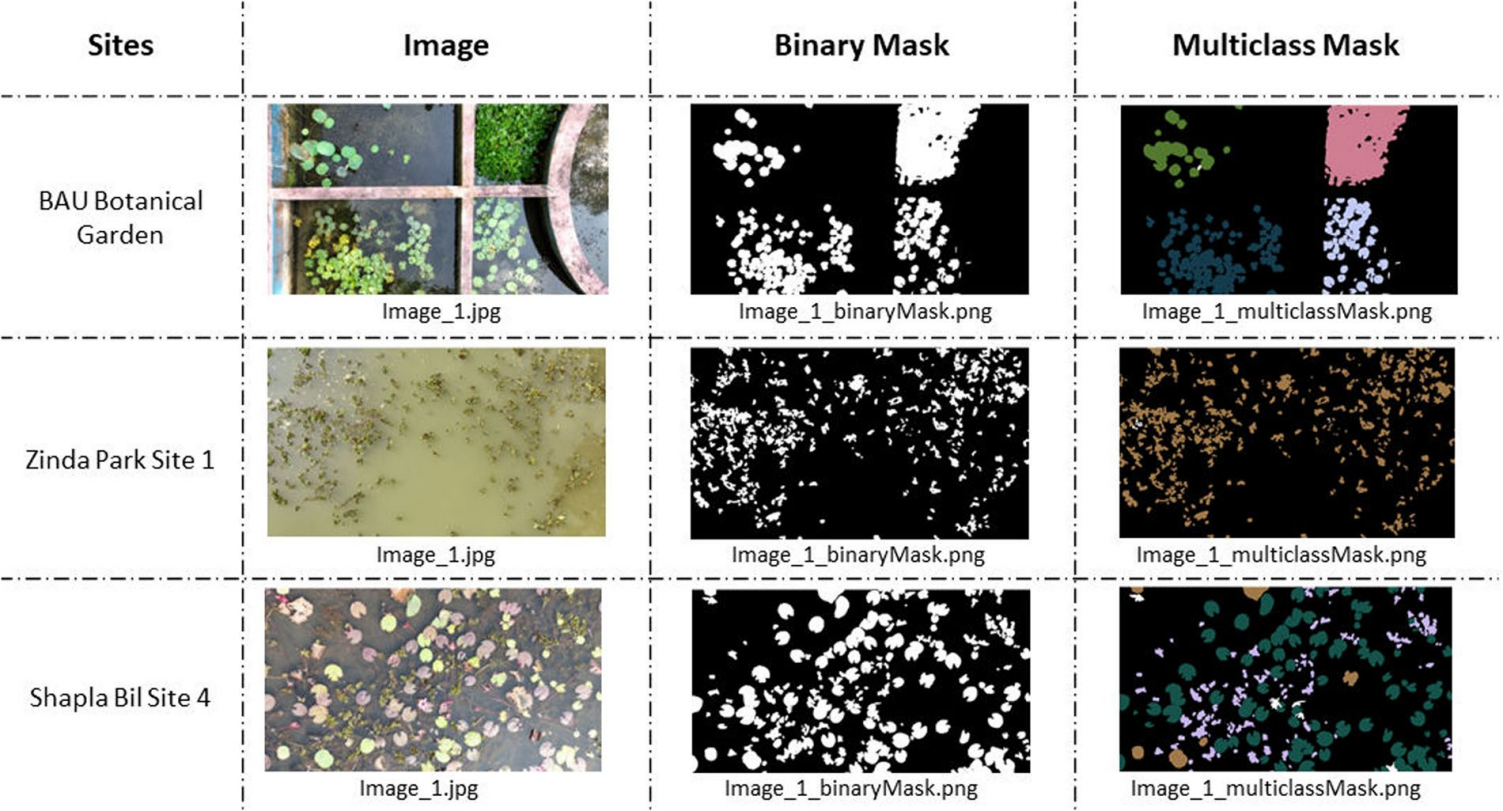
Main image, binary and multiclass ground truth mask in different sites.

In this step, the segmentation mask for each image is produced from the mask outlining. These segmentation masks are the ground truth for the dataset. Both binary and multiclass semantic segmentation masks are created. In binary segmentation, the image is segmented into two parts, foreground and background. For this dataset, the foreground designates the image area covered by all the plants, and the background designates water or submerged land area. To produce a binary mask, each pixel in an image is annotated with 1 if it represents a plant or 0 for the background. In Fig. 4 column 3 samples of binary masks generated for three images are shown.

In multiclass segmentation, an image is segmented into different regions based on different features. In this case, each aquatic plant class represents a feature in the image. Therefore, pixels in an image denoting plants of the same class are annotated with the corresponding class number (according to Table 2). For example, Lily Pad (Scientific name: *Nymphaea spp*) and Water Chestnut (Scientific Name: *Trapa natans*) are assigned to numeric class levels 3 and 9, respectively (see Table 2). If an image contains these two plants, then all pixels representing Lily Pad are annotated with the numeric value 3 and all pixels representing Water Chestnut are encoded with the numeric value 9. In Fig. 4 column 4 shows sample of multiclass masks generated for three images. Consequently, each image in the dataset is complemented with two segmentation masks (binary and multiclass) in png format. Several scripts are written using the PIL, numpy, os and cv2 libraries in Python 3.0 to perform this step.

**Fig. 5:**
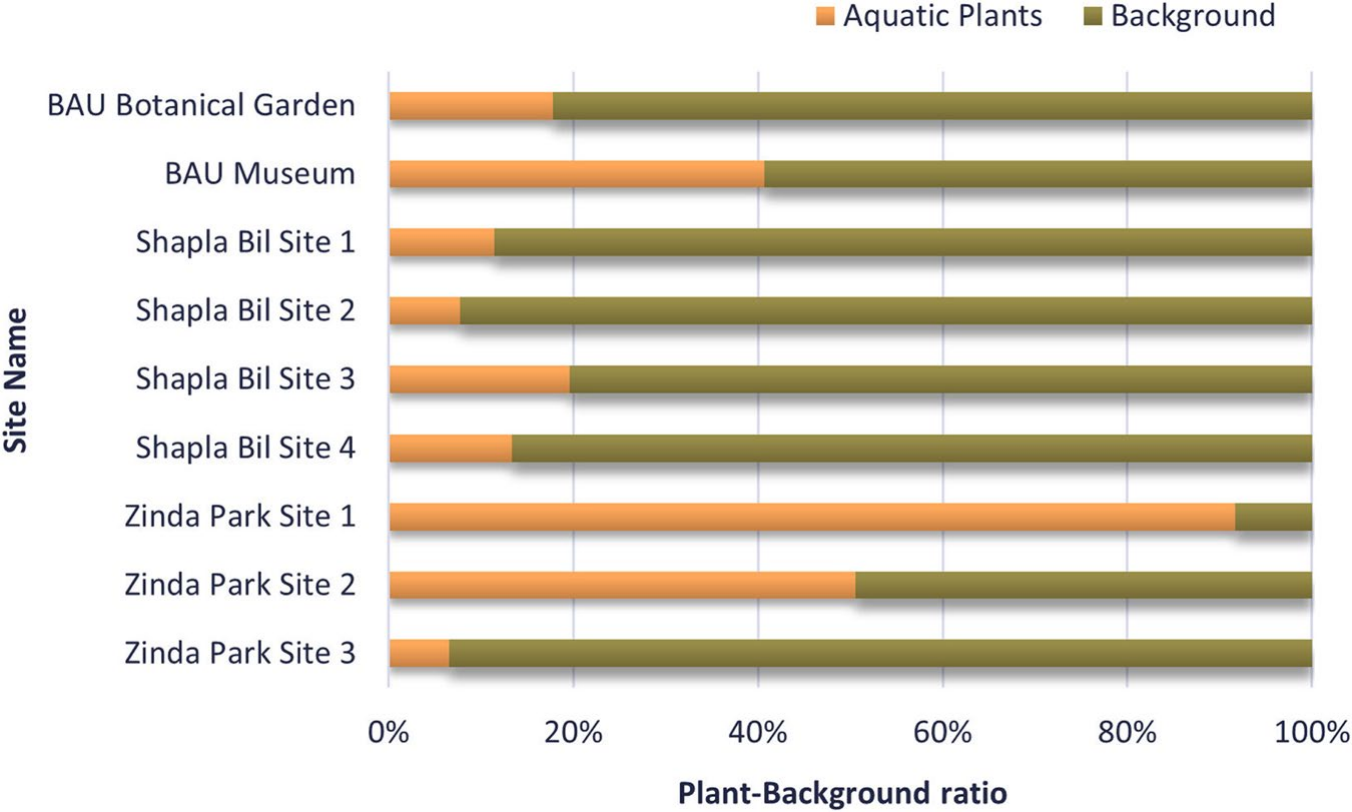
Aquatic plant ratio in binary semantic segmentation.

The binary segmentation mask is often used to estimate the density of aquatic plants^28^. In the dataset, the foreground of a binary mask represents the plant pixels and the background designates the water body. This information can be used in an area measurement algorithm to estimate the ratio of plants in an image^29^. Figure 5 shows the ratio of aquatic plants for all 9 (nine) data collection sites. It can be seen that Site 1 in Zinda Park has a plant ratio of 90% compared to Site 3 having only 6.5%. This assessment is fundamental for measuring the intensity of light interception by aquatic plants, tracking photosynthetic activity, and overall biomass production in a given site, among others.

The multiclass segmentation can be applied for a detailed survey of the shape, size, health, and population ratios of specific aquatic plants^1^. The multiclass segmentation masks presented in column 4 in Fig. 4 show the classes of aquatic species that are observed at the given locations. By accurately distinguishing these classes and their distribution, researchers can detect indigenous and invasive species, measure possible imbalances in plan growth to preserve biodiversity, and prevent extinction^30^. Furthermore, pixel level investigation can assist disease detection, growth monitoring, and track plant behavior^1^.

**Fig. 6:**
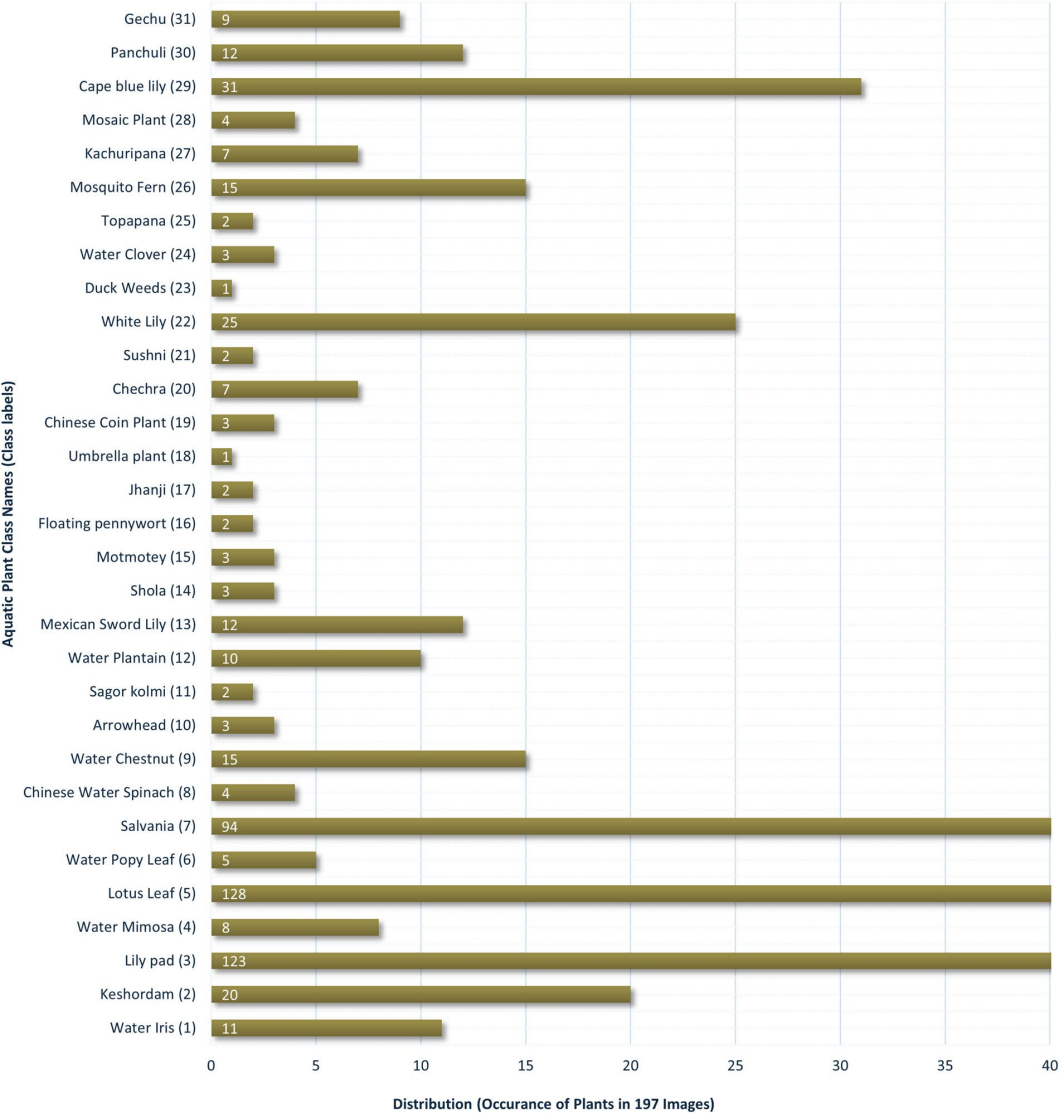
Plant distribution of the AqUavplant dataset^20^.

Finally, the distribution of aquatic plant species across the dataset is measured, and the observed result is presented in Fig. 6. In this figure, the y-axis listed the 31 species classes and the x-axis presents the total number of occurrences in 197 images. It is evident that the class distribution is imbalanced, with few species like Lily Pad (Class 3) occurring in 123 images, while the rare species, such as Umbrella Plant (Class 18) and Duck Weed (Class 23), are found in single image only. This disproportionate ratio of instances between different species classes may lead to biased models (ML/DL) that misclassify the minority classes and result in poor classification performance. Modern data imbalance handling methods such as bilateral branch network^31^, weighting based on effective sampling^32^, algorithm-level adjustments, cost-sensitive learning, and anomaly detection^33^ can be applied to balance the dataset. Furthermore, the synthetic augmentation technique^34^ can also be used to handle the under represented classes. In addition, the authors of this paper are currently working on acquiring drone images of the rare species from several locations in Bangladesh. Upon selection, verification, and preparation, the image data will be contributed to the dataset, maintaining the balance distribution.

## Data Records

**Fig. 7:**
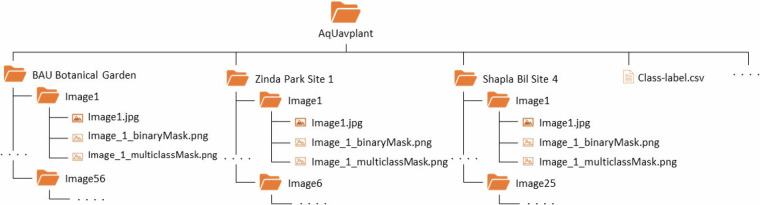
Folder structure of the AqUavplant dataset^20^.

The *AqUavplant* dataset, available on Figshare^20^, is distributed under the CC BY 4.0 license, permitting reuse without restriction. The organizational structure of the image and ground truth musk folders is depicted in Fig. 7. The parent folder, AqUavplant, includes 9 sub-folders, each corresponding to a distinct data collection site (identified by their respective folder names). Within each site-specific sub-folder, images are sorted into individual image folders. Each of these image folders contains three distinct image files: the original captured image (jpg format), a binary segmentation mask (png format), and a multiclass segmentation mask (png format). A uniform naming convention is followed for all images to ensure consistency and facilitate easy access (as visualized in Fig. 7). The dataset encompasses 197 images representing 31 species of aquatic plants, all captured at 4K resolution (3840 px  × 2160 px). Additional metadata is available in the *Class-label.csv* file, which detail each of the 31 species classes and their associated class levels (integer values ranging from 0 to 31) used for generating the segmentation masks.

## Technical Validation

To validate the quality and applicability of the dataset, several cutting edge deep learning models are implemented for semantic segmentation using the dataset. The reported results are interpreted accordingly using dice coefficient, jaccard index, and accuracy metrics. To enhance model performance, multiple data splitting strategies are adopted in addressing the data imblance issue.

### Dataset Splits

The dataset^20^ is divided into train, validation, and test sets with a ratio of 70:10:20, respectively. To handle the imbalance in the dataset, the split is done in two categories, namely location-based and stratified. In location-based data split, images acquired from the BAU Botanical Garden and BAU Museum (see Fig. 2) are used as a train dataset and images from other seven sites are used as a validation and test dataset. This split tentatively balances the distribution of aquatic plant classes as the majority of plants are present in the BAU location (as shown in Table 2). In stratified data split, images are partitioned to train, validation, and test sets according to the ratio, as some classes have fewer samples than others and are not available in all locations (see Table 2 and Fig. 6). This selective approach to data partitions also ensures the presence of most classes of aquatic plants in the data splits. However, few plant classes (e.g., classes 11, 16, 17, 18 in Fig. 6) are present in one or two images only; therefore, they are kept in the training set only.

### Models and training details

Eight state-of-the-art semantic segmentation models are implemented, namely, U-Net^35^, R2U-Net^36^, Attention U-Net^37^, R2AU-Net^38^, DeepLabV3^39^, DeepLabV3+^40^ and Swin Transformer^41^, and their performance is evaluated based on the dataset. All the models are run for 100 epochs on a device equipped with NVIDIA T4 GPU. The 100 epochs are chosen as the validation loss begins to stabilize after this point, indicating that further training will not significantly improve the performance of the model. To reduce computational complexity, the image size is set to 512 px  × 512 px. This compression is lossy but imperative due to the memory constraint, as considering the original resolution while processing images would exceed our available GPU resources and the score may vary with different image sizes.

The U-Net is an encoder-decoder-based network primarily designed for localization tasks. It is a U-shaped architecture consists of a contracting path (encoder) that extracts essential features and an expansive path (decoder) that reconstructs a detailed localization mask. The performance of U-Net is improved by integrating additional layers (e.g., residual skip connections, recurrent convolutional layers, and transpose convolutional layers) that resolve the vanishing gradient problem of the U-Net. This improved version is named as the recurrent residual (R2) U-Net. Another variation of U-Net is proposed by adding attention gates on the decoder side, known as the attention U-Net. This integration helps the attention U-Net to investigate the most relevant features to increase the overall performance. Following this track, the novel features of attention U-Net and the R2U-Net are integrated to develop R2AU-Net. Other segmentation model, e.g., DeepLabV3 utilizes atrous (dilated) convolution to increase context awareness. The Atrous Spatial Pyramid Pooling (ASPP) module of this model captures multi-scale features without altering the dimension of the feature map. DeepLabV3 is further enhanced by integrating a decoder module that refines the segmentation results on object boundaries, popularly known as DeepLabV3+. Finally, the Swin Transformer segmentation model a hierarchical, window-based attention mechanism to efficiently capture both local details and global context in images, enhancing its ability to understand complex structures and relationships. Flexibility and efficiency make it a strong contender in modern computer vision applications.

### Evaluation Metrics

To evaluate the performance of the models, the following metrics are used: Dice coefficient, Jaccard index, F1 score and the Accuracy score. The dice coefficient and the Jaccard index (also known as *IoU*) check the overlap between the ground truth mask and the prediction mask. The accuracy (*Acc*) is used to measure the percentage of correctly identified pixels for each class. A weighted F1 score is used for multiclass segmentation due to class imbalance in the dataset^42^. Eq. (1) defines all four metrics.

1 $$\begin{array}{c}\\ Acc=\frac{{T}_{p}+{T}_{n}}{{T}_{p}+{F}_{p}+{T}_{n}+{F}_{n}}\\ F1=\frac{2\cdot {T}_{p}}{2\cdot {T}_{p}+{F}_{p}+{F}_{n}}\\ Dice=\frac{2\times {T}_{p}}{({T}_{p}+{F}_{p})+({T}_{p}+{F}_{n})}\\ Jaccard=\frac{{T}_{p}}{{T}_{p}+{F}_{p}+{F}_{n}}\end{array}$$

Where, *T*_*p*_, *T*_*n*_, *F*_*p*_, and *F*_*n*_ denote True positive, True negative, False positive and False negative, respectively.

### Evaluation Results and Analysis

The model performance benchmark of the AqUavplant dataset^20^ is reported for both binary and multiclass semantic segmentation in Tables 3 and 4, respectively, considering results of stratified and location-wise splits. It is noticed that the pre-trained weight (trained on 20 categories of COCO dataset^43^- available on PyTorch) boosts the performance of DeepLabV3 and outperforms other models in stratified and location-wise splits. Considering results of the binary segmentation in Table 3 accuracy of all models surplus 80% but the class detection accuracy remains low because of low values of the Dice Coefficient and Jaccard Index which vary from 42 to 69 and 38 to 56, respectively, while considering the stratified split. Similarly, for the location-wise split the maximum accuracy is 85.90% for the R2AU-Net and the dice coefficient is 52. 83% along with the 38. 94% Jaccard index for the pre-trained DeeplabV3.

**Table 3:** Binary segmentation benchmarking on different segmentation models.

Model Name	Split					
	Stratified			Location-wise		
	Acc.	Dice Coeff.	Jaccard Index	Acc.	Dice Coeff.	Jaccard Index
U-Net^35^	87.89	58.17	43.78	84.97	43.46	38.84
R2U-Net^36^	84.70	59.59	44.47	85.06	50.45	35.47
Attention U-Net^37^	86.37	64.19	50.49	84.93	45.63	31.27
R2AU-Net^38^	85.19	63.77	49.88	**85.90**	46.00	31.95
DeepLabV3^39^	89.35	59.37	46.25	85.18	50.10	35.43
DeepLabV3+^40^	82.35	48.13	46.39	82.42	48.16	46.45
SwinTransformer^41^	77.15	42.26	38.57	77.97	42.01	38.98
DeepLabV3 (pretrained)^39^	**91.38**	**69.22**	**56.49**	83.20	**52.83**	**38.94**

**Table 4:** Multiclass segmentation bench-marking on different segmentation models.

Model Name	Split							
	Stratified				Location-wise			
	Acc	F1 score	Dice Coeff.	Jaccard Index	Acc	F1 score	Dice Coeff.	Jaccard Index
U-Net^35^	**85.07**	**84.11**	44.79	37.42	79.30	74.28	26.29	23.43
R2U-Net^36^	80.99	80.09	41.30	34.59	**79.73**	76.66	30.56	26.54
Attention U-Net^37^	81.66	82.13	42.37	35.10	79.37	74.42	26.52	23.62
R2AU-Net^38^	80.18	78.74	40.43	34.15	78.41	76.81	31.04	27.18
DeepLabV3^39^	82.02	83.01	45.22	37.77	79.47	76.89	35.59	29.11
DeepLabV3+^40^	80.76	02.88	02.88	02.66	77.64	03.04	03.04	02.96
SwinTransformer^41^	77.15	69.70	24.01	22.14	77.97	71.93	22.90	21.20
DeepLabV3 (pretrained)^39^	84.09	83.50	**50.79**	**42.92**	79.59	**77.66**	**36.89**	**31.21**

**Fig. 8:**
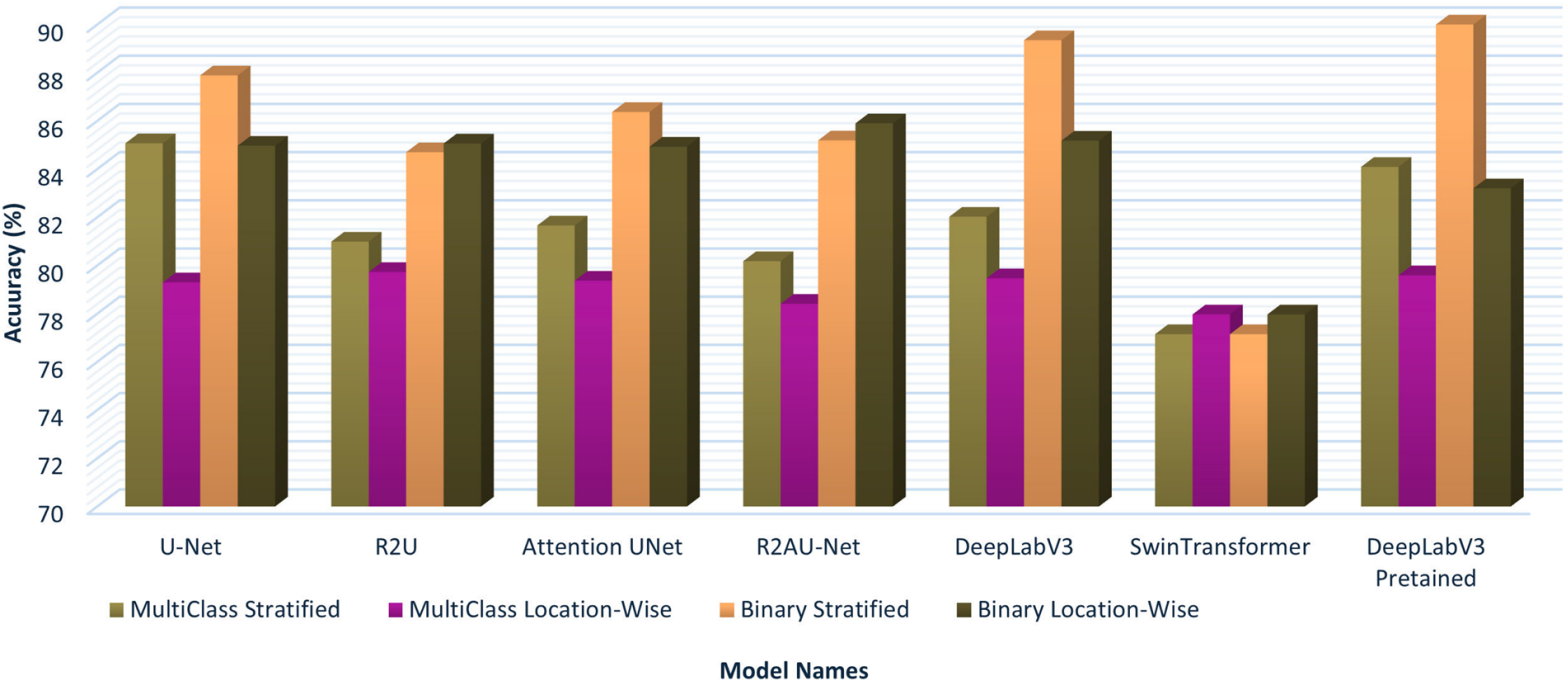
Accuracy comparison of different segmentation models for the AqUavplant dataset.

Multiclass semantic segmentation results are illustrated in Table 4. Due to the large number of classes and class imbalance, the performance of multiclass semantic segmentation is less satisfactory. Pre-trained DeepLabV3 has shown the best performance among all models. Moreover, weighted F1 scores are also low because of class imbalance where U-Net achieves a score of 0.61% higher than that of the pre-trained DeepLabV3 in the stratified split, but the pre-trained DeepLabV3 outperforms all other models in the location-wise split. Recent model like Swin Transformer face challenges with binary and multiclass classifications. However, with proper tuning and class balancing techniques, performance is expected to improve^44^. U-Net outperforms all other models with 85.07% accuracy and 84.11% F1 score in the stratified split. For the location-wise split, the pre-trained DeepLabV3 has shown the most prominent score with 79.59% accuracy and 36.89% F1 score. The summary of binary and multiclass segmentation performance on both splits is illustrated in Fig. 8.

## Usage Notes

- *AqUavplant*^20^ dataset consists of 197 high resolution (3840px  × 2160px, 4K) images of 31 aquatic plant species collected from 9 (nine) different sites in Bangladesh. The DJI Mavic 3 Pro triple-camera professional drone is used with a GSD value of 0.04-0.05 cm/px for optimal image collection without losing detail.
- There are several uses of the dataset in the domain of agriculture, botany, and environmental science. For example, measuring the intensity of light interception and overall biomass production by aquatic plants, and tracking photosynthetic activity at a given site. Researchers can detect the diversity of indigenous and invasive species, monitor plant growth and diseases, measure the growth ratio to preserve biodiversity, and prevent extinction^30^. Water quality indicators, water habitat possibilities, and water treatment policies can be developed by analyzing the dataset.
- The assessment of eight semantic segmentation models on the dataset indicates encouraging outcomes for both binary and multiclass segmentation tasks. Subsequent studies should focus on advancing the effectiveness of multiclass semantic segmentation algorithms, taking into account the species diversity and the inherent dataset imbalance.
- The disproportionate ratio of instances in the dataset among various species classes undermines ML/DL models. This can lead to misclassification in minority classes and degrade overall classification accuracy. To address data imbalance, modern techniques such as bilateral branch networks^31^, effective sampling-based weighting^32^, algorithm-level adjustments, cost-sensitive learning, and anomaly detection^33^ can be employed to methodically balance the dataset. Moreover, synthetic data augmentation techniques^34^ are also applicable. Additionally, the research team is collecting drone images of rare species from diverse locations in Bangladesh, which will be added to the dataset, thereby enhancing its balance distribution.

## Data Availability

All the codes for data loading and model run for the bench-marking codes are available at https://github.com/aia39/AqUavplant-Dataset. Users are suggested to adopt the customized PyTorch-based data loader for new models or approaches.
